# Divergent SARS-CoV-2-specific T cell responses in intensive care unit workers following mRNA COVID-19 vaccination

**DOI:** 10.3389/fimmu.2022.942192

**Published:** 2022-10-06

**Authors:** Estefanía Salgado Del Riego, María Laura Saiz, Viviana Corte-Iglesias, Blanca Leoz Gordillo, Cristina Martin-Martin, Mercedes Rodríguez-Pérez, Dolores Escudero, Carlos Lopez-Larrea, Beatriz Suarez-Alvarez

**Affiliations:** ^1^ Servicio de Medicina Intensiva, Hospital Universitario Central de Asturias, Oviedo, Spain; ^2^ Instituto de Investigación Sanitaria del Principado de Asturias (ISPA), Oviedo, Spain; ^3^ Translational Immunology, Instituto de Investigación Sanitaria del Principado de Asturias (ISPA), Hospital Universitario Central de Asturias, Oviedo, Spain; ^4^ Servicio de Microbiología, Hospital Universitario Central de Asturias, Oviedo, Spain; ^5^ Translational Microbiology, Instituto de Investigación Sanitaria del Principado de Asturias (ISPA), Hospital Universitario Central de Asturias, Oviedo, Spain; ^6^ Servicio de Inmunología, Hospital Universitario Central De Asturias, Oviedo, Spain

**Keywords:** SARS-CoV-2, specific T-cell response, intensive care unit workers, vaccination, ELISpot, CD4^+^ T cells, CD8^+^ T cells

## Abstract

The cellular immune response to severe acute respiratory syndrome coronavirus 2 (SARS-CoV-2) in response to full mRNA COVID-19 vaccination could be variable among healthy individuals. Studies based only in specific antibody levels could show an erroneous immune protection at long times. For that, we analyze the antibody levels specific to the S protein and the presence of SARS-CoV-2-specific T cells by ELISpot and AIM assays in intensive care unit (ICU) workers with no antecedents of COVID-19 and vaccinated with two doses of mRNA COVID-19 vaccines. All individuals were seronegative for the SARS-CoV-2 protein S before vaccination (Pre-v), but 34.1% (14/41) of them showed pre-existing T lymphocytes specific for some viral proteins (S, M and N). One month after receiving two doses of COVID-19 mRNA vaccine (Post-v1), all cases showed seroconversion with high levels of total and neutralizing antibodies to the spike protein, but six of them (14.6%) had no T cells reactive to the S protein. Specifically, they lack of specific CD8^+^ T cells, but maintain the contribution of CD4^+^ T cells. Analysis of the immune response against SARS-CoV-2 at 10 months after full vaccination (Post-v10), exhibited a significant reduction in the antibody levels (p<0.0001) and protein S-reactive T cells (p=0.0073) in all analyzed individuals, although none of the individuals become seronegative and 77% of them maintained a competent immune response. Thus, we can suggest that the immune response to SARS-CoV-2 elicited by the mRNA vaccines was highly variable among ICU workers. A non-negligible proportion of individuals did not develop a specific T cell response mediated by CD8^+^ T cells after vaccination, that may condition the susceptibility to further viral infections with SARS-CoV-2. By contrast, around 77% of individuals developed strong humoral and cellular immune responses to SARS-CoV-2 that persisted even after 10 months. Analysis of the cellular immune response is highly recommended for providing exact information about immune protection against SARS-CoV-2.

## Introduction

Since the first vaccine against SARS-CoV-2 was approved by the regulatory agencies and millions of people around the world were vaccinated, the pandemic has been analyzed from a different perspective. New questions arising about such matters as the long-term effectiveness of the vaccines, the number of doses or boosters needed, and how interindividual variability is affected, have only been partially answered. We know that mRNA vaccines, in particular Pfizer-BioNTech (BNT162b2,Comirnaty) and Moderna (mRNA-1273, Spikevax), provide up to 95% protection against COVID-19 (1, 2), but the level of specific neutralizing antibodies against the S protein diminish over time. Consequently, a third booster dose, even fourth, is being administered to the most vulnerable population, including aged people and health care workers, as well as to the general population (3, 4).

Most of the relevant studies done so far have been based on antibody levels but it remains partially unclear whether the individuals who have received two doses of vaccine have developed a long-term protective cellular response against SARS-CoV-2 and, more important still, whether all healthy people are adequately immunized after these two doses. To investigate this, we focus on a cohort of COVID-19 intensive care unit (ICU) workers, who are one of the groups who have been at high risk of infection from the start of the pandemic until the present time. The seroprevalence of SARS-CoV-2 antibodies among these workers has been extensively analyzed, not only to measure the effectiveness of vaccines since they were one of the earliest groups to be vaccinated, but also to ensure their safety and the success of the measures adopted to contain the infection against new variants (5).

Studies in healthcare workers, who are comparable to other healthy individuals, showed that vaccination induces higher antibodies levels in people previously exposed to SARS-CoV-2, and that one dose is enough to produce the maximum antibodies levels and to maintain them for up to 1 year (6). However, in unexposed healthy workers, two doses are required to obtain robust humoral immunity, and this declines over time, leading to the advocation of the administration of a booster vaccine shot (7). In relation to the cellular immune response, numerous studies have evaluated its strength and durability after mRNA vaccination in healthy people (8–13), but also in older people (14, 15), or in patients with some immunodeficiencies (16–19). Studies in unexposed healthy people showed that a robust humoral and cellular response is triggered in response to second vaccination, although first-dose mRNA vaccination is enough to trigger the immunological memory in COVID-19-recovered subjects (20, 21). Combined analysis of SARS-CoV-2 mRNA vaccines revealed a coordinated immune response mediated by a rapid antigen-specific CD4^+^ T cell response followed by a gradual development of CD8^+^ T cells more variable in magnitude (22). Specifically, circulating T follicular helper (Tfh) cells represent a key fraction of specific CD4^+^ T cells being crucial for the development of memory B cells, plasma cells and support antibody response following vaccination (23). First studies with BNT162b1 showed that two doses are required to elicit a robust CD4^+^ (100% responders) and CD8^+^ T (85% responders) cell response, with a favorable Th1 profile that enhances the quality of cytotoxic cells (12). Spike-specific CD4^+^ (100% responders) and CD8^+^ T (87% responders) cells responses peaked after the second dose of the mRNA-1273 vaccine and were largely maintained up to 6 months after vaccination, with a decline mainly in CD8^+^ T cells (9). Memory spike-specific CD8^+^ T cells produce mainly IFN-γ and co-express granzyme B exhibiting an effector memory surface phenotype. Oberhardt V et al. (24) showed that these vaccine-elicited CD8^+^ T cells are early mobilized, one week after the first dose, when CD4^+^ T cells and antibodies are undetectable and undergo a robust expansion after the second dose generating a pool of highly differentiated CD8^+^ T cells with a relevant effector function. Recently, it has also been reported that the frequency of stem cell-like memory (T_SCM_) cells one-two weeks post-second vaccination determinates the longevity of memory CD8^+^ T cells induced by SARS-CoV-2 mRNA vaccines (25). SARS-CoV-2-reactive T and B cells persist over time even as the levels of antibodies decline, suggesting that the vaccines do provide durable protection against severe disease (26) and against new variants (27). However, we cannot discount the possibility that interindividual variability might lead to heterogeneous immune responses that will condition the durability of the protection from SARS-CoV-2 infection and COVID-19 disease, even in individuals who are not of older ages or immunocompromised (28).

The aim of this study was to establish the genuine degree of protection against SARS-CoV-2 in ICU workers who have been highly exposed to the virus, and to determine how long the protection lasts after vaccination. Using the T ELISpot and flow cytometry activation induced marker (AIM) assays, we analyzed the SARS-CoV-2- T cells that are reactive against the main structural viral proteins —spike (S), membrane (M) and nucleocapsid (N) — before vaccination, to establish the influence of a pre-existing response, and 1 and 10 months after full vaccination with mRNA vaccines, to analyze the durability of the anti-viral immune response and the requirement for additional boosters.

## Results

### Pre-existing T cells against SARS-CoV-2 proteins in highly exposed ICU workers

**Figure 1:**
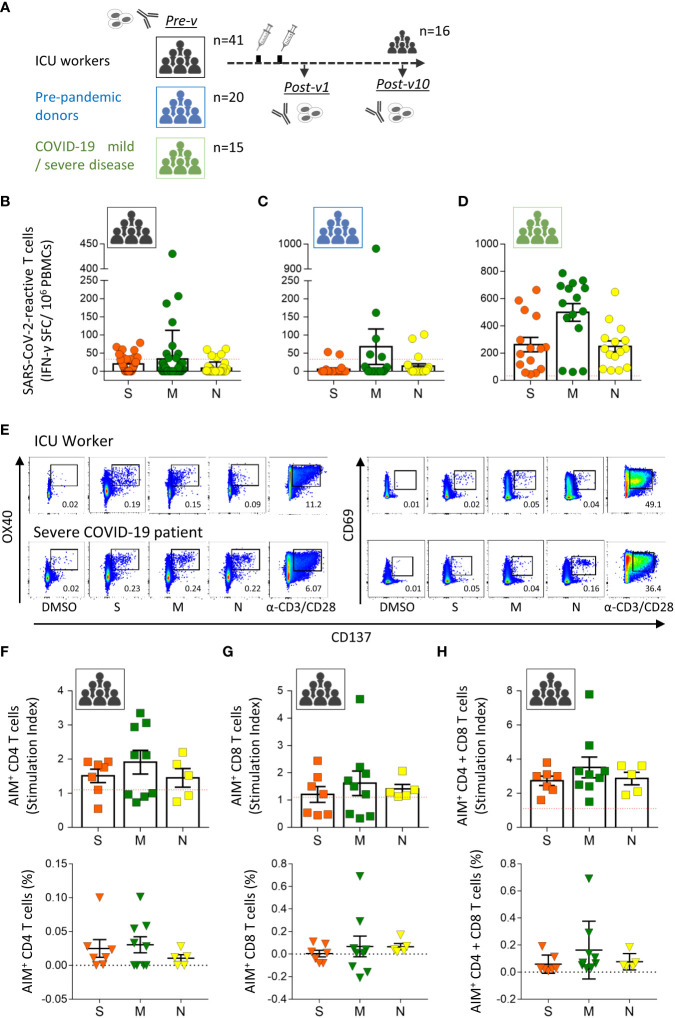
Pre-existing SARS-CoV-2-specific T cells in healthy ICU workers. **(A)** Study design. Blood samples were taken from intensive care unit workers with no known antecedents of COVID-19 before vaccination (n=41, Pre-v), and 1 month (n=41, Post-v1) and 10 months (n=16, Post-v10) after receiving two doses of the mRNA vaccine. Pre-pandemic healthy donors (n=20) and COVID-19 convalescent patients (n=15) with mild and severe disease were used as control groups. Antibody levels and SARS-CoV-2-reactive T cells were assayed at the indicated times. Frequencies of IFN-γ-producing T cells assayed by ELISpot assay against spike (S, orange circles), membrane (M, green circles) and nucleocapsid (N, yellow circles) proteins in samples from ICU workers **(B)** taken before vaccination, pre-pandemic healthy donors **(C)** and convalescent patients recovered from COVID-19 **(D)**. Data from ELISpot assays are depicted as the number of spot-forming cells (SFCs) per 1 x 10^6^ PBMCs. The red dashed line shows the established cut-off (≥33.3 SFCs/10^6^ cells) after subtraction of negative control values. **(E)** Flow cytometry dot plots from one ICU workers showing the representative gating strategy to identify antigen specific CD4^+^ (CD3^-^ CD4^+^ OX40^+^ CD137^+^) and CD8^+^ (CD3^-^ CD8^+^ CD69^+^ CD137^+^) T cells by AIM assay. A patient with severe COVID-19 was reference as positive control. DMSO and polyclonal induction with anti-CD3/CD28 antibodies were used as negative and positive controls of stimulation for each sample. Numbers in dot plots represent the percentage of AIM^+^ cells in each indicated square. Number of CD4+ **(F)**, CD8^+^ **(G)** T cells or both **(H)** detected by AIM assay against spike (S, orange squares, n=7), membrane (M, green squares, n=9) and nucleocapsid (N, yellow squares, n=5) proteins in samples from ICU workers that were positive by ELISpot assay. Data from AIM assay are represented as stimulation index (SI, squares) and frequency (triangles). Red dashed line represents the limit of positivity for SI > 1.1. All data are represented as mean ± SEM.

**Figure 2:**
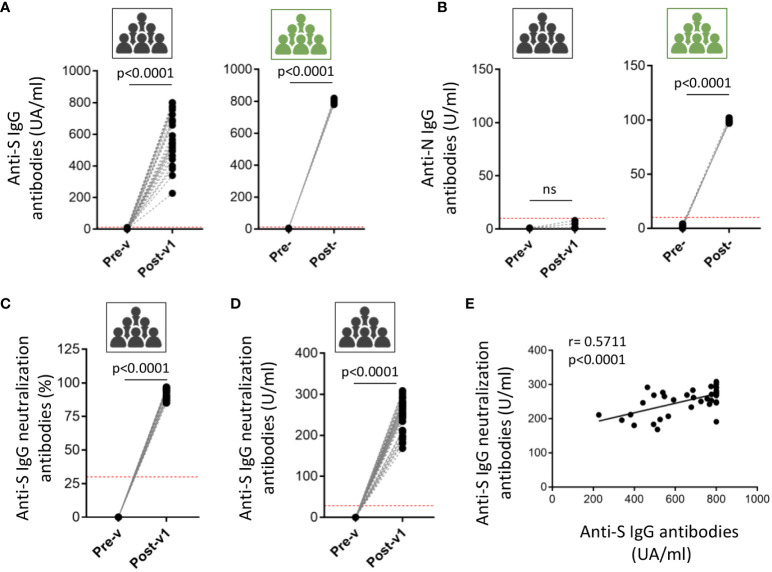
Induced humoral response in ICU workers after COVID-19 mRNA vaccination. The antibodies titer against full-length spike (S) protein **(A)** and nucleocapsid (N) protein **(B)** was quantified in serum samples from ICU workers (black icons) at times previous to vaccination (Pre-v) and one month after full vaccination (Post-v1), and from five COVID-19 convalescent patients with severe disease (green icons) determined before (Pre-) and after (Post-) infection. Neutralizing antibodies were measured in serum samples from ICU workers (black icons) at times previous to vaccination (Pre-v) and one month after full vaccination (Post-v1) and data are represented as the inhibition percentage **(C)** and quantified by units per ml (U/ml) **(D)**. Red dotted-lines in panels **(A-D)** mark the limit of positivity. Statistical comparisons were performed by Wilcoxon test. **(E)** Correlation between frequencies of the titers of neutralizing and total anti-S IgG antibodies in ICU workers one month after full vaccination. Statistical by Spearman correlation coefficient. ns, not significant.

The primary aim of this study was to analyze the presence of SARS-CoV-2-specific T cells in COVID-19 ICU workers who had been highly exposed to the virus during the first (March 2020) and second (November 2020) pandemic waves in Spain and who remained unvaccinated (**Figure 1A**). None of the participants (n=41) had a history of SARS-CoV-2, COVID-19 disease, or household contact with SARS-CoV-2-positive people (**Table 1**). Initially, we determined the number of SARS-CoV-2-specific memory T cells by using the ELISpot assay to determine the IFN-γ-producing cells. For that, PBMCs were stimulated with overlapping peptides pools spanning the three main structural proteins of SARS-CoV-2, the spike (S), membrane (M) and nucleocapsid (N) proteins. We found 14 of the 41 individuals (34.1%) to have pre-existing T lymphocytes specific for at least one of the three analyzed SARS-CoV-2 structural proteins (**Figure 1B**). The dominant target of the pre-existing SARS-CoV-2-specific T cells was the M protein (detected in 9/41 of the samples; 22.0%), followed by the S protein (7/41; 17.1%) and the N protein (5/41; 12.2%) (**Figure 1B**). The distribution of these specific T cells varied considerably between individuals: some showed reactivity against one or two proteins, and two individuals reacted against all three SARS-CoV-2 proteins (**Supplementary Figure 1A**). Moreover, we determined the presence of antibodies specific to the S and N proteins (**Figure 2A**), showing absence of both antibodies in all individuals and corroborating the lack of asymptomatic COVID-19.

**Table 1:** Characteristics of individuals groups under study.

	ICU workers	Pre-pandemic	Mild/Severe COVID-19
N° of participants; n	41	20	15
Male/Female; n	9/32	12/8	12/3
Age; median ± SD	39 ± 12.31	40 ± 12.09	62 ± 9.08
SARS-CoV-2 infection by RT-PCR, n (%)	0	0	15 (100%)
**Comorbidities, n (%)**			
Heart disease	0	0	2 (13.3)
Diabetes Mellitus	1 (2.4)	0	4 (26.6)
Hypertension	0	0	10 (66.6)
Cancer	0	0	0
Pulmonary disease	0	0	5 (33.3)
**Medications, n (%)**			
Statins	3 (7.3)	–	4 (26.6)
Levothyroxine	6 (14.6)	–	0
ARA II	0	–	9 (60)
Immunosuppressant	0	–	0

(-) not determined.

To elucidate whether the presence of pre-existing T cells against SARS-CoV-2 proteins are due to the continuous exposition to the virus in COVID-19 ICUs, we assayed a cohort of 20 healthy donors obtained in pre-pandemic time, between 2007 and 2013 years (**Table 1**). A total of 7 out of 20 (35%) pre-pandemic donors showed reactivity and respond to the viral proteins with a similar frequency to the one detected in ICU workers (**Figure 1C**). Again, the specific T cell response against the M protein was the majority (25% of samples) followed by the one against the N (15%) and S proteins (10%), suggesting a major role of the M protein in the development of cross-reactivity against other coronaviruses. To further understand the magnitude of the cellular immune response developed after exposition to the SARS-CoV-2 virus, we compared the levels of these pre-existing T cells with the observed in COVID-19 convalescent patients with mild or severe infection (**Table 1**). All patients were diagnosed by PCR and have S- and N-reactive IgG antibodies. The development of specific T lymphocytes against the three structural proteins of the virus was detected in all COVID-19 patients at high levels, indicating that the cellular response triggered under virus exposition was clearly higher than the one observed in unexposed individuals, ICU workers and pre-pandemic donors (**Figure 1D**).

In order to corroborate the results obtained by ELISpot assay and discriminate the contribution of SARS-CoV-2-specific CD4^+^ and CD8^+^ T cell responses, we performed AIM assay in samples obtained from ICU workers. The gating strategy used and representative flow cytometry plots of one ICU worker and one COVID-19 patient is showed in **Supplementary Figure 2** and **Figure 1E**. We detect the presence of antigen-specific T cells in all analyzed samples, being in some individuals mediated by CD4^+^ T (**Figure 1F** **)** cells and by CD8^+^ T (**Figure 1G**) lymphocytes in others, but no correlation between them was observed for any protein (**Supplementary Figure 1B**). However, when the contribution of CD4^+^ and CD8^+^ T cells for each individual was added, all individuals showed a positive response (**Figure 1H**), and the distribution of positive specific T cells was identical between ELISpot and AIM assays (**Supplementary Figure 1C**), showing the utility of both methods to detect pre-existing T cells against SARS-CoV-2 and their effector ability secreting IFN-γ. As we have previously reported by ELISpot assay, the magnitude of cellular response determined by AIM assay was slightly higher against M protein, and mainly mediated by CD4^+^ T cells.

Altogether, some ICU workers showed a specific cellular response against SARS-CoV-2 proteins, at similar levels to pre-pandemic donors, probably because of the cross-reactivity between SARS-CoV-2 and other coronaviruses or viruses, and not by the continuous exposition to the virus in the COVID-19 units. That pre-existing immunity is associated with the contribution of both CD4^+^ and CD8^+^ T cells, but variable among individuals.

### Divergent cell-mediated immune response induced by SARS-CoV-2 mRNA vaccination

Following these results, the humoral and cellular immune responses were analyzed in the same individuals 1 month (Post-v1) after receiving two doses of COVID-19 vaccine, with the aim of determining whether the pre-existing immunity might condition the immune response to the vaccines. Only the reactivity against the S protein could be analyzed since all the approved mRNA COVID19 vaccines have been designed to act on the S protein. To corroborate the effectiveness of vaccination, we measured seroconversion, and found that all individuals developed high levels of anti-S protein antibodies, with titers >200 AU/ml in all cases, although the levels varied among samples (**Figure 2A**). Moreover, SARS-CoV-2 nucleocapsid-reactive IgG was not detected in any sample showing that no asymptomatic COVID19 infection was experimented by these ICU workers during the time of the study (**Figure 2B**). As positive control group, we determine the presence of anti-S and anti-N immunoglobulins in five COVID19 patients, being all the detected values upper than the superior limit of the assays (**Figures 2A, B**). Besides, we analyzed the presence of neutralizing antibodies using a surrogate virus neutralization assay based on the ability of the antibodies to neutralize the RBD (receptor binding domain of the spike protein)-ACE2 interaction. Results are shown as the percentage of neutralization potential (**Figure 2C**) and the antibodies titers (**Figure 2D**). In both cases, we observed the induction of high levels of neutralizing antibodies in all ICU workers after one moth having received the full vaccination. Moreover, a significant correlation between the titers of neutralizing and anti-S IgG antibodies (**Figure 2E**) suggest that most of the antibodies induced after vaccination are able to block the virus entry.

**Figure 3:**
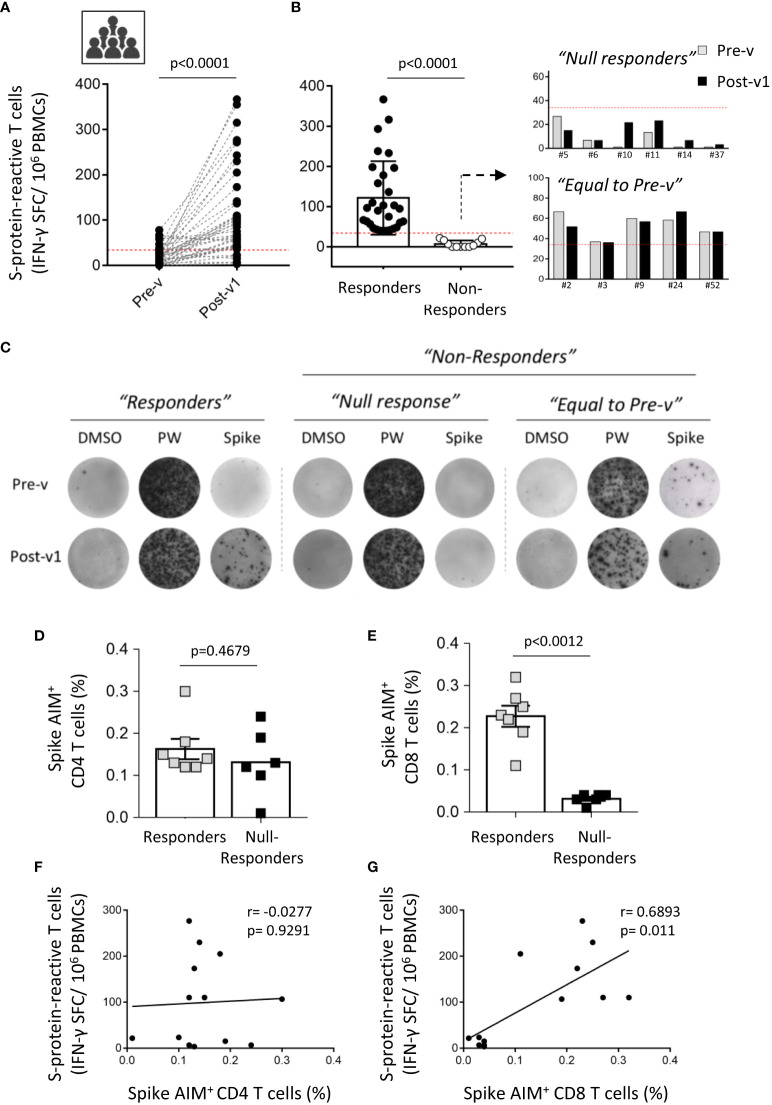
Interindividual variation of the cellular immune response to SARS-CoV-2 after COVID-19 mRNA vaccination. **(A)** Paired graph showing the number of specific T cells reactive against the S protein was evaluated by ELISpot assay in PBMCs from ICU workers (n=41) obtained before vaccination (Pre-v) and 1 month (Post-v1) after full vaccination. Statistical comparisons were performed by Wilcoxon test. **(B)** Frequency of spike-specific T cells detected by ELISpot assay in samples with a positive response (“Responders”) o those with a null response (“Non-responders”). The latter were divided in “Null responders” when the number of specific T cells was below the cut-off (<33.3 SFCs/10^6^ PBMCs) and “Equal to Pre-v” when the number of specific T cells is not enhanced after vaccination. **(C)** Representative images of IFN-γ-inducing spots in “Responders” and “Non-responders” groups. **(D, E)** Percentage of spike AIM^+^ CD4^+^ and CD8^+^ T cells in seven individuals from “Responders” group and six samples that lack of cellular response to SARS-CoV-2 (“Null responders” group). Statistical comparisons were performed by two tail Mann Whitney test. **(F, G)** Correlation between frequencies of spike AIM^+^ CD4^+^ and CD8^+^ T cells and the number of spike-reactive T cells evaluated by ELISpot assay in the individuals corresponding to “Responders” (n=7) and “Null responders” (n=6) groups. Statistical was performed by Spearman correlation coefficient.

Next, we analyzed the presence of S-protein-specific T cells by ELISpot assay and most of the individuals developed a strong cellular response; IFN-γ-Spot Forming Cells (SFC), Pre-v: 19.8 ± 20.64; Post-v1: 108 ± 97.86; p<0.0001 (**Figure 3A**). Surprisingly, we observed that six individuals (6/41; 14.6%) did not develop any SARS-CoV-2-reactive T cells showing a number of S-protein-reactive T cells below of the established detection limit (SFC ≤ 33.3/10^6^ of PBMCs) (**Figure 3B**). These individuals were named “Null responders”. Moreover, to analyze the cellular immune response specific against SARS-CoV-2 only as consequence of vaccination, we compared the number of pre-existing specific-T cells against the S protein (Pre-v) from the number of S protein-reactive T cells obtained 1 month after vaccination (Post-v1). We observed that of the seven individuals with pre-existing T cells against S protein, five showed similar levels of SARS-CoV-2-specific T cells to those they had before vaccination (**Figure 3B**), suggesting there was no increased response to vaccination (“Equal to Pre-v” group), and only two developed a stronger cellular immune response after vaccination, despite the presence of pre-existing T cells. Together, these results indicated that whereas 30 individuals (73.2%, named “Responders”) developed a moderate or elevated number of S-specific T cells, 11 (26.8%), showed a number of specific-T cells below the established cut-off or at equal levels before vaccination (named “Non-responders”) (**Figures 3B, C**). Thus, nearly one out of four healthy individuals do not reach a strong and effective cellular immunity against SARS-CoV-2 after receiving two doses of the vaccine, despite the high titer of specific antibodies developed.

According to this, no significant correlation was found between the humoral and cellular immune responses assayed by ELISpot assay one month after full vaccination (**Supplemental Figure 3A**). Moreover, the absence of SARS-CoV-2-specific T cells was not associated with any clinical parameter, treatment or diagnosed immune condition in these ICU workers (data not shown). However, patient chronological age was significantly correlated with the antibody titer, although not with the cellular immune response (**Supplemental Figures 3B, C**). Younger ICU workers showed a higher titer of S protein-specific antibodies. These correlations were similar when neutralizing antibodies were taken into account, showing a significant correlation with age but not with the cellular response (**Supplemental Figures 3D, E**).

To corroborate the results found in the cellular response by a different method, we performed AIM assay in seven samples from “Responders” and six samples from “Null responders” groups. Unexpectedly, we detect that both ICU workers groups developed AIM^+^ CD4^+^ T cells (“Responders”: 0.1629 ± 0.0241; “Null-responders”: 0.1317 ± 0.0322; p=0.4679) (**Figure 3D**), whereas only “Responders” individuals showed a strong AIM^+^ CD8^+^ T cell-mediated response cells (“Responders”: 0.2271 ± 0.0249; “Null-responders”: 0.03167 ± 0.0047; p=0.0012) (**Figure 3E**). That is supported by the significant correlation observed between S-protein reactive T cells assayed by ELISpot and spike AIM^+^ CD8^+^ T cells (p=0.011), and not with spike AIM^+^ CD4^+^ T cells (p=0.9291) (**Figures 3F, G**). Moreover, in these analyzed individuals, no significant correlation between AIM^+^ CD4+ T cells and total IgG and neutralizing antibodies titers was observed (data not shown). Thus, these results suggest that the absence of cellular response detected in the “Null responders” group by ELISpot assay could be consequence of the lack of a specific immune response against the S protein mediated by CD8^+^ T cells, the main effector cells producing IFN-γ.

### An early immune response might condition the durability of the immunological memory to SARS-CoV-2

**Figure 4:**
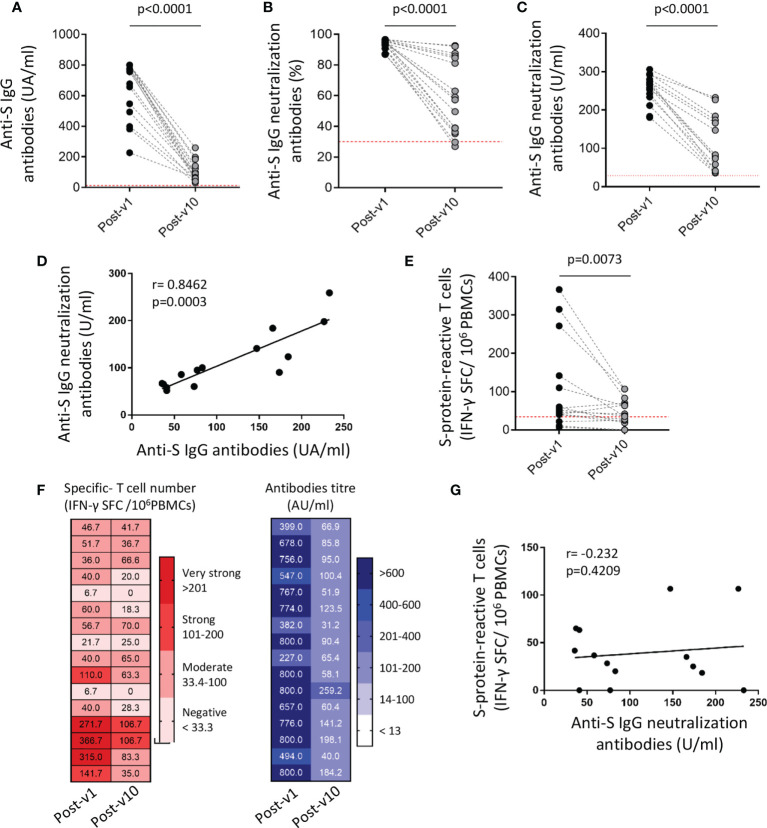
SARS-COV-2-specific humoral and cellular immune responses decline over time but persist 10 months after vaccination. The titer of anti-S IgG and neutralizing antibodies **(A–C)**, the correlation between them **(D)** and the frequency of S protein-specific T cells **(E)** were assayed in blood samples (n=16) obtained 1 month (Post-v1) and 10 months (Post-v10) after full vaccination. **(F)** Global distribution of the number of specific T cells (left) and total S IgG titers (right) in samples (n=16) obtained at 10 months (Post-v10) after receiving the second dose of the vaccine, and comparison with the values obtained at 1 month (Post-v1) after vaccination. Numbers indicate the S-specific T cells number and antibodies titers and each line represents one ICU worker. **(G)** Correlation between neutralizing antibodies and the number of spike-reactive T cells in ICU workers at 10 months after full vaccination. Statistical comparisons were performed by Wilcoxon test and correlation with Spearman correlation coefficient.

Subsequently, the persistence of the long-term immune response to SARS-CoV-2 was assayed in a small number of individuals (n=16), from the collection of samples 10 months after full vaccination (Post-v10). Overall significant decreases in anti-spike immunoglobulins (p<0.0001), percentage (p<0.0001) and titer of neutralizing antibodies (p<0.0001) in plasma samples were observed in all individuals (**Figures 4A–C**). As expected, no anti-nucleocapsid antibodies were observed in ICU workers (data not shown), because of mRNA vaccines are aimed against spike protein and no one was infected with SARS-CoV-2 during this time after vaccination. The specific antibody levels declined in the long term, but any samples became seronegative during this time. Again, the correlation between total anti-spike IgG and neutralizing antibodies was highly significant (p=0.0003; **Figure 4D**), suggesting that the decrease in the number of specific antibodies goes in hand with the reduction in their neutralizing ability. When the cellular response was analyzed, we also observed a significant contraction or reduction of the specific T cell number over time (p=0.0073) (**Figure 4E**). The comparison of the humoral and cellular immune responses detected at ten months after vaccination (Post-v10), revealed that all individuals showed a reduction in the antibody and specific T cells levels, but maintain neutralizing antibodies levels upper than the detection limit. Moreover, only three individuals showed a decline in the specific-T cell count to levels below the cut-off compared with the number of specific T cells elicited at one month after vaccination (**Figure 4F**). On the other hand, 76.9% (10/13) of individuals with a positive cellular response after 1 month still had a competent immune response 10 months after receiving their second dose. As expected, individuals (n=3) who lacked positive T cells against the S protein one month after vaccination (Post-v1) remained negative. Despite that both humoral and cellular immune responses are decreased, again no correlation between them was observed (**Figure 4G**).

In conclusion, most of the individuals maintain a stable repertoire of T cells and antibodies specific to SARS-CoV-2 ten months after having received full vaccination, and those with the highest levels of specific antibodies and cells one month after vaccination are the ones who experienced the least reduction over time. Although more studies are required to ensure strong conclusions, our results provide insights that the immune response reached initially after vaccination could condition the durability of an effective immune response.

## Discussion

Although progress continues to be made to understand the humoral and immune responses arising from the administration of COVID-19 mRNA vaccines, many questions remain unanswered and may only be resolved with time and as the pandemic naturally evolves. In the meantime, a range of scenarios need to be analyzed, taking into account the diversity among individuals and the immune history of each. Here, we analyzed the immune response against SARS-CoV-2 over time in healthy individuals, COVID-19 intensive care unit workers who were not infected during the first or second waves of the pandemic in Spain and who received two doses of vaccine by February/March 2021.

The main findings of our study were that: (i) before vaccination, and in the absence of specific antibodies, 34.1% of individuals had T cells that reacted against some of the main structural proteins of SARS-CoV-2, mainly the M protein, and remarkably, this percentage resembles to that one detected in pre-pandemic healthy donors; (ii) all individuals showed high neutralizing antibody titers 1 month after full vaccination, although 14.6% of them had not developed any S-specific T cells by ELISpot assay; (iii) when the contribution of CD4^+^ and CD8^+^ T cells was independently analyzed, we observe a lack of cellular response mediated by CD8^+^ T cells in these “Null responders” individuals; (iv) there were clear long-term declines in the humoral and cellular immune responses, although negative seroconversion was not detected in any individual 10 months after full vaccination, and of greater relevance, still 76.9% of the individuals with a positive cellular response at 1 month after vaccination showed values of specific T cells above the limit of positivity at long-term; and (v) no correlation between humoral and cellular immune responses was observed at any time after full vaccination. Thus, our findings indicate that in healthy individuals whose immune system responded properly to the vaccination against the SARS-CoV-2 S protein, the humoral and cellular immunity that developed early on are specific and durable; however, a small but far from negligible proportion of people, around 15%, did not develop a T cell response, regardless of their age or known history of immunodeficiency.

Several studies have reported the existence of SARS-CoV-2-reactive T cells in SARS-CoV-2-unexposed healthy people (29–34). Braun and colleagues (35) reported that around 35% of seronegative SARS-CoV-2-unexposed healthy donors had CD4^+^ T cells that were reactive mainly against the C-terminal portion of the SARS-CoV-2-S protein, although this was a lower proportion than in COVID-19 patients. Similarly, in our cohort of ICU healthy workers, around 34% of the samples showed T cells reactive against SARS-CoV-2 proteins, and the number of specific T cells was considerably reduced compared to the observed in COVID-19 convalescent patients. Additional studies of geographically diverse cohorts reported that 20-50% of healthy donors unexposed to SARS-CoV-2 had detectable levels of specific T cells against structural (S, M, N) and non-structural (ORF1a and ORF1b) proteins of SARS-CoV-2 (32). In our study, and according to these previous results, SARS-CoV-2-specific T cells mainly directed against the M protein were detected in SARS-CoV-2-seronegative ICU workers.

Initially, we postulated that the continuous exposition of ICU workers to patients with severe COVID-19 could trigger a slight but efficient cellular immune response specific to SARS-CoV-2 that might influence the response against further SARS-CoV-2 infections. However, the presence of pre-existing T cells in ICU workers was similar to the one detected in samples obtained from healthy donors before the pandemic, who were not exposed to the SARS-CoV-2. Thus, we guess that the origin of these cross-reactive T cells against SARS-CoV-2 proteins is not generated by the virus itself, but the possible recognition of SARS-CoV-2 epitopes shared with other seasonal viruses as it has been shown in previous studies (36, 37). One of the hypotheses suggested is that these unexposed individuals could have developed a variable, short-lived antibody response to another coronavirus but supplemented by a more sustained cellular immune response. Whereas the humoral cross-immunity is weak and decay rapidly (38), the cross-reactivity cellular immunity persists and contributes to SARS-CoV-2 immune responses upon infection or vaccination. Accordingly, SARS-CoV-2-reactive T cells respond to the restimulation with peptide pools obtained from other common coronaviruses (229E and OC43), which is evidence of their origin as an earlier immune response to an endemic human coronavirus (39). However, additional studies using different MHC-II epitopes reported that the high frequency of these cells in unexposed individuals cannot be completely explained by the homology among seasonal coronaviruses, and even that some might be naive and respond to unrelated pathogens (32). In our study, both crossreactive CD4+ and CD8+ T cells subsets were detected. Moreover, there are some discordances between the diverse reported studies. For instance, we and others observed a higher prevalence of pre-existing T cells with cross-reactive against membrane protein, whilst other studies showed the cellular response to the nucleoprotein as the most prevalent. These differences could be due to the varied geographical origin of the studied cohorts and the differential exposition to diverse human coronavirus.

The presence of pre-existing cross-reactive T cells has been suggested to contribute to the variation in COVID-19 disease outcome (40, 41), increase the strength of the immune response in SARS-CoV-2 infection and vaccination (42), or even give rise to new therapeutic options that could be built upon and used as passive cell immunotherapy (43). But until now, few studies have evaluated whether these pre-existing T cells contribute to the host defense against SARS-CoV-2 or conversely, impair the development of an effective immune response. Only studies evaluating the severity of SARS-CoV-2 infection in individuals with pre-existing SARS-CoV-2-specific T cells will allow assessing the truly biological relevance of these cells. Meanwhile, Loyal et al. (18) identified a peptide (S816-830) located within the fusion peptide domain of spike protein that is recognized by CD4+ T cells in 20% of the unexposed individuals. Most of the individuals, but not all, increased the frequency of cross-reactive T cells after infection or vaccination, suggesting their reactivation and role to mediate the secondary response. By contrast, we showed that only two of the seven ICU workers enhanced the frequency of pre-existing T cells after vaccination, while five maintained similar levels to the ones detected before vaccination though always above the limit of positivity stablished. Therefore, we postulated that cross-reactive T cells can be generated by the previous exposure to diverse human coronaviruses but only some generated against specific peptides might be boosted upon SARS-CoV-2 infection or vaccination. However, a better phenotypical and functional characterization of pre-existing cross-reactive T cells versus a newly induced SARS-CoV-2-specific T cell response is required to further comprehend their differences and in consequence, whether they could modulate the severity and outcome of the disease.

Following the immune response upon SARS-CoV-2 vaccination, we observed high S protein and neutralizing IgG titers in all individuals only 1 month after complete administration as a consequence of the high efficacy of SARS-CoV-2 mRNA vaccines (1, 2). Antibody levels decline was slightly associated with age, although we should point out that there were no elderly people in our cohort. However, we detected strong variability in the cellular immune response triggered after full vaccination. It is quite remarkable that six (14.6%) individuals, all under 40 years of age, had not developed a cellular response against the S protein. COVID-19 disease is characterized by the great variability of its severity, from asymptomatic to severe, or in the worst cases, with a fatal outcome. In addition, however, the magnitude of the innate and adaptive immune responses to SARS-CoV-2 among people could condition the immune response to further infections, variants or to the vaccination (44). Thus, it is worth considering immunological heterogeneity in all situations, rather than just in the transplant, autoimmunity, and oncological contexts in which immune dysregulation is clearly established by the received therapy. One of the first studies reported by Sahin U et al. (12) showed that most of the vaccinated individuals, but not all, developed Th1-skewed immune responses and IFN-γ-producing CD4^+^ and CD8^+^ T cells. However, not all individuals responded to the vaccination, remaining the interindividual variability in response to vaccination poorly understood, whereas most studies have tried to understand the durability of the immunological memory. Multiple factors could condition the T cell response to vaccination or to any new viral infection, such as the repertory of naive T cells, which declines substantially with age but also with the persistent activation of T cells by other chronic viruses, such as cytomegalovirus; deficient ability of cytotoxic T cells to induce an effector response mediated by IFN-γ and cytolytic granules production or the induction of antigen-specific regulatory T cells under subimmunogenic conditions (45), among others.

Our study was designed to analyze for differences in the overall cellular immune response to vaccines, but the specific contribution mediated by CD4^+^ or CD8^+^ T cells was also determined. Cassaniti I et al. (46) showed that the overall SARS-CoV-2 specific T-cell response in convalescent patients was reduced to about 95% and 80% after CD4 or CD8 T-cell depletion, respectively. By using activation assays and cytokine production determinations, other studies showed that upon SARS-CoV-2 exposition the immune responses mediated by CD4^+^ cells are predominant, but SARS-CoV-2-specific CD8^+^ T cells were also reported (47). Most of the studies are commonly carried out upon infection and not after vaccination, and some differences could be observed compared to natural infection although are not fully understood. Goel RR et al. (8) described that after the second dose of SARS-CoV-2 mRNA vaccine, all individuals generated high levels of CD4^+^ T cells, regardless of any prior SARS-CoV-2 infection, and most of them produced a CD8^+^ T cell response. After an initial contraction, these specific memory T cells stabilized and began to decline but remain at least 6 months after vaccination. In addition, the early CD4^+^ T cells response detected in those individuals correlated with the intensity of humoral immunity at long-term. Likewise, in our study, the humoral and cellular responses to SARS-CoV-2 weakened by 10 months after administration of the complete vaccine regime. Nonetheless, we do not detect such correlation neither at 1 month or 10 months after vaccination. On the other hand, we observed that those patients with the highest responses after 1 month of vaccination, undergo a lessened reduction in the antibody titer and specific T cells number, suggesting that the intensity of the early immune response after vaccination may condition their durability and that a third, booster dose is not required so early for all people. Initially, there was a little controversy about whether mRNA vaccines generated CD8+ T cell responses and at what time were produced. Oberhardt et al. (24) reported that CD8^+^ T cells are early generated after vaccination, even when antibodies and CD4^+^ T cells are scarcely detectable and there are highly differentiated effector CD8^+^ T cells. These cells remain stable and fully functional at long-term and together with antibodies and CD4^+^ T cells act in coordination to maintain a full protection. Thus, a combined response mediated by both, CD4^+^ and CD8^+^ T cells, is triggered after vaccination and required to confer protection at different times. In this respect, we observed that the six ICU workers who does not developed IFN-γ-producing specific T cells 1 month after vaccination, are the same that lacked AIM^+^ CD8^+^ T cells. However, they do maintain a modest cellular response mediated by CD4^+^ T cells, like the one detected in the “Responders” ICU workers group with a positive determination by ELISpot assay. Thus, our results suggest that early after vaccination, CD8^+^ T cells are mostly contributing to the IFN-γ production. In this sense, it has been previously reported that the time of antigen exposure required to trigger effector cells is different between CD4^+^ and CD8^+^ T cells, showing these latter a faster rate of cell division and a lower threshold of activation (48). Moreover, we cannot rule out that the length of peptides used for the technical approaches could be also conditioning the MHC-I or MHC-II presentation, although this needs further confirmation.

We recognize that our study is limited by the relatively small number of individuals considered. It would be desirable to have more subjects to enable firm conclusions to be drawn, but to our knowledge, that is one of the scary studies evaluating the complete immune protection (humoral and cellular immunity) against SARS-CoV-2 in highly exposed COVID-19 ICU workers. Although our results demonstrated no correlation between the humoral and cellular responses after vaccination, it should be required to analyze the frequency of Tfh cells to understand the direct association between these cells and the neutralizing antibodies, such as it has been reported by Vikkurthi R et al. (49) with BBV152 vaccine in an Indian cohort. Unfortunately, this kind of studies require a greater number of PBMCs that were not available in our samples. Our results nevertheless suggest that the pattern of the cellular immune response to SARS-CoV-2 after vaccination is highly variable among healthy individuals. Some individuals lack an effective cellular response after receiving two doses of vaccine, but others may be sufficiently protected 10 months after vaccination and so might not require a booster dose. Although we demonstrated that the absence of IFN-γ-producing T cells is mainly mediated by CD8^+^ T cells, an exhaustive functional characterization of these cytotoxic T cells could help to understand the lack of specific cellular response in these individuals.

In summary, after receiving two-doses of COVID-19 vaccine, a strong humoral immune response is produced in all individuals, but the cellular immune response, mainly mediated by CD8^+^ T cells, is more variable. This supports the notion that it exists an interindividual variability to SARS-CoV-2 vaccination among healthy people that could condition their protection at long-term and thus, determining the SARS-CoV-2-specific T cell response might be of great value not only for establishing real immune competence after vaccination, but also for scheduling subsequent booster doses in highly exposed healthy workers, given that the cellular immune response can be detected in most individuals even 10 months after full vaccination.

## Materials and methods

### Population, samples and data collection

The observational and prospective study includes COVID-19 ICU workers (n=41, 26 nurses and 15 doctors) at the Central University Hospital of Asturias (Oviedo, Spain), with no antecedents of COVID-19 according to previous COVID-19 symptoms and household contacts. Moreover, we analyzed the humoral and cellular immune response against SARS-CoV-2 proteins in 20 healthy blood donors collected before pandemic (pre-pandemic group) and 15 convalescent patients with mild or severe COVID-19 disease diagnosed by viral RT-PCR test on respiratory samples. The demographic and clinical characteristics of the three groups are provided in **Table 1**.

Whole blood was collected from all individuals in appropriate collection tubes, serum samples were stored at -80°C until analysis, and peripheral blood mononuclear cells (PBMCs) were isolated by Ficoll (Lymphoprep) density-gradient centrifugation using standard protocols and frozen, maintaining their viability, until use. Samples from ICU workers group were collected between December 2020 and November 2021, specifically, one-week before SARS-CoV-2 vaccination (Pre-v) and then about 1 month (4-5 weeks) after their second dose (Post-v1) (**Figure 1A**). In 16 patients, an additional sample was taken 10 months after full vaccination (Post-v10). All participants received vaccination with Comirnaty (BNT162b2) except one, who received Spikevax (mRNA1273). Pre-pandemic samples were collected in two times, during 2007 and between December 2012 and January 2013. Another groups of COVID-19 convalesvent individuals was diagnosed by viral RT-PCR test and all required hospitalization.

All participants gave their written informed consent for inclusion. The study was approved by the ethic committee of research of Principality of Asturias (CEImPA, n° 2020.521 “*Study of the cellular immunity against SARS-CoV-2 in high-risk healthy workers*”) and informed consent was obtained from all participants in compliance with the Helsinki Declaration of 1975

### SARS-CoV-2-specific antibodies

Serum samples were tested for SARS-CoV-2 antibodies against the S protein using an automated commercial chemiluminescent system on the LIAISONXL^®^ platform. The LIAISON^®^ SARS-CoV-2 TrimericS IgG assay (DiaSorin, VC, Italy) was used to quantify IgG antibodies to the anti-trimeric spike glycoprotein of SARS-CoV-2. This test has a clinical sensitivity of 98.7% and specificity of 99.5%, and a good correlation with microneutralization test results (PPA: 100% and NPS: 96.9%). Results are presented in arbitrary units per ml (AU/ml), with a cutoff of 13 AU/ml, and a maximum response of 800 AU/ml. Antibodies against nucleocapsid protein were determined using the BioPlex2200 SARS-CoV-2 IgG panel (Biorad) following the manufacturer´s instructions. This test has a clinical sensitivity of 96.3% and specificity of 99.8% and results are shown in units per ml (U/ml), being values ≤ 9 U/ml considered negative and positive for ≥10 U/ml.

For detection of neutralizing antibodies, we used the cPass SARS-CoV-2 Neutralization Antibody Detection Kit (Genscript) following the manufacturer´s instructions. This test allows to determinate the ability of antibodies to block the interaction of the SARS-CoV-2 receptor-binding domain (RBD) and the human ACE2 receptor. Samples were diluted 1:10 and the percentage of inhibition was determined using the formula: (1-OD value of sample/OD value of negative control) x 100%. Samples were run by duplicate and percentage of inhibition below 30% were considered as no detectable neutralizing antibodies. Additionally, we used the SARS-CoV-2 neutralizing antibody calibrator (Genscript) to generate a calibration curve and show the semi-quantitative results as units per ml (U/ml). Values ≥ 28.6 U/ml were considered positive.

### SARS-CoV-2-reactive T cells detection by ELISpot

The cellular immune response against SARS-CoV-2 was evaluated by the ELISpot assay using the anti-IFN-γ ELISpot kit (AID^®^ GmbH, Strasberg, Germany) to measure counts of IFN-γ- producing T cells that had previously been stimulated with SARS-CoV-2 peptide pools. To achieve this, PBMCs (3 x 10^5^/well) in AIM-V medium (Gibco, MA, USA) were cultured for 16-18 h with the specific antigens of interest on plates precoated with an anti-IFN-γ antibody. Overlapping peptide pools (15-mers with an 11-amino acid overlap) against the SARS-CoV-2 S (ref 130-126-700), M (ref 130-126-702) and N (ref 130-126-698) proteins (all from Miltenyi Biotec, Bergisch Gladbach, Germany) were used as a stimulus at a concentration of 0.5µg/ml. Pokeweed mitogen (AID GmbH), with high mitogenic activity on T and B lymphocytes, and AIM-V medium were used as positive and negative controls, respectively. Plates were developed according to the manufacturer’s protocol and spot-forming cells (SFCs) were read with an AID iSpot reader system using AIS ELISpot version 7.0 software (AID GmbH, Germany). Samples were assayed in duplicate, and results were obtained as the mean count of spots after subtracting the frequency with medium alone, and expressed as the number of SFCs per million PBMCs. T cell response was considered positive when mean spot counts were >10 SFCs per well or ≥33.3 spots/10^6^ cells after subtracting the negative control frequency.

### SARS-CoV-2-reactive T cells determination by activation-induced markers

The activation-induced markers (AIM) assay was performed as previously described (50, 51) to determine the antigen-specific CD4^+^ and CD8^+^ T cells. For that, PBMCs were thawed in AIM-V medium (Gibco, MA, USA) and stimulated for 24 hours in the presence of SARS-CoV-2 specific megapools (1µg/ml). The megapools consist of peptide pools of 15-mers overlapping by 11-amino acid and aimed against the SARS-CoV-2 S (ref 130-126-700), M (ref 130-126-702) and N (ref 130-126-698) proteins (Miltenyi Biotec). An equimolar concentration of DMSO and a mix of anti-CD3 (3µg/ml) and anti-CD28 (1µg/ml) antibodies were used as negative and positive controls, respectively. After stimulation, cells were washed with PBS containing 2% FBS and 0.05 mM EDTA and further surface stained with a cocktail of antibodies for 1 hour at 4°C in the dark. The following antibodies were used for multiparametric flow cytometry: CD3 APC-Cy7 (SK7), CD8 BV605 (HIT8a), CD137 APC (4B4-1), CD69 PE-Cy7 (FN50), OX40 PE (ACT35) and CD4 PerCP (SK3). All antibodies were purchased from Biolegend, CA, USA. The DNA-binding dye, DAPI (0.1 µg/ml, Santa Cruz Biotechnologies, TX, USA) was used for live/dead discrimination. After staining, cells were washed and resuspended in FACS buffer for further acquisition using Cytek^®^ Aurora 3L Spectral Analyzer (Cytek Biosciences). Data were analyzed using FlowJo version 10.8.1. The percentage of antigen-specific CD4^+^ and CD8^+^ T cells was calculated by subtracting the DMSO percentages, set as background. Stimulation Index (SI) was calculated as the ratio between the percentage of AIM^+^ cells after stimulation with peptide pools and the percentage of AIM^+^ cells after DMSO stimulation, and SI > 1.1 was considered positive.

### Statistical analysis

Data are expressed as the mean ± standard error of mean (SEM). Associations between variables were assessed by Spearman correlation and comparisons between samples using Fisher´s exact test, Wilcoxon paired-samples test, or Mann-Whitney test for comparison of unpaired samples. Statistical analyses were carried out with IBM SPSS Statistics v20.0 (Armonk, NY, USA) and GraphPad-Prism v7 (San Diego, CA, USA). Statistical significance was concluded for values of p<0.05. Statistical details of the experiments and significance are noted in the respective figures and figure legends.

## Data availability statement

The original contributions presented in the study are included in the article/**Supplementary Material**. Further inquiries can be directed to the corresponding author.

## Ethics statement

The studies involving human participants were reviewed and approved by CEImPA, 2020.521 “Study of the cellular immunity against SARS-CoV-2 in high-risk healthy workers”. The patients/participants provided their written informed consent to participate in this study.

## Author contributions

Conceptualization, BS-A and CL-L; design of the work, BS-A; ESdR; MLS; acquisition of samples, ESdR; CM-M; DE; and BLG; methodology, BS-A; MLS; VC-I; MR-P; interpretation of data and analysis BS-A; MLS and DE; writing the manuscript BS-A; ESdR and VC-I; revision and agree of the manuscript, all authors; funding acquisition BS-A and CL-L. All authors contributed to the article and approved the submitted version.

## Funding

This study was supported by the Plan Nacional de I+D+I 2013-2016 ISCIII (Spanish Institute of Health Carlos III; grant numbers PI19/00184 and PI20/00639) and Gobierno del Principado de Asturias, PCTI-Plan de Ciencia, Tecnología e Innovación 2021-2023 (Grant number IDI/2021/000032).

## Conflict of interest

The authors declare that the research was conducted in the absence of any commercial or financial relationships that could be construed as a potential conflict of interest.

## Publisher’s note

All claims expressed in this article are solely those of the authors and do not necessarily represent those of their affiliated organizations, or those of the publisher, the editors and the reviewers. Any product that may be evaluated in this article, or claim that may be made by its manufacturer, is not guaranteed or endorsed by the publisher.
