# Supplementary figures and images for: Divergent SARS-CoV-2-specific T cell responses in intensive care unit workers following mRNA COVID-19 vaccination
Source: Front Immunol. 2022 Oct 6;13:942192. doi: 10.3389/fimmu.2022.942192 (PMC9582956; doi:10.3389/fimmu.2022.942192)

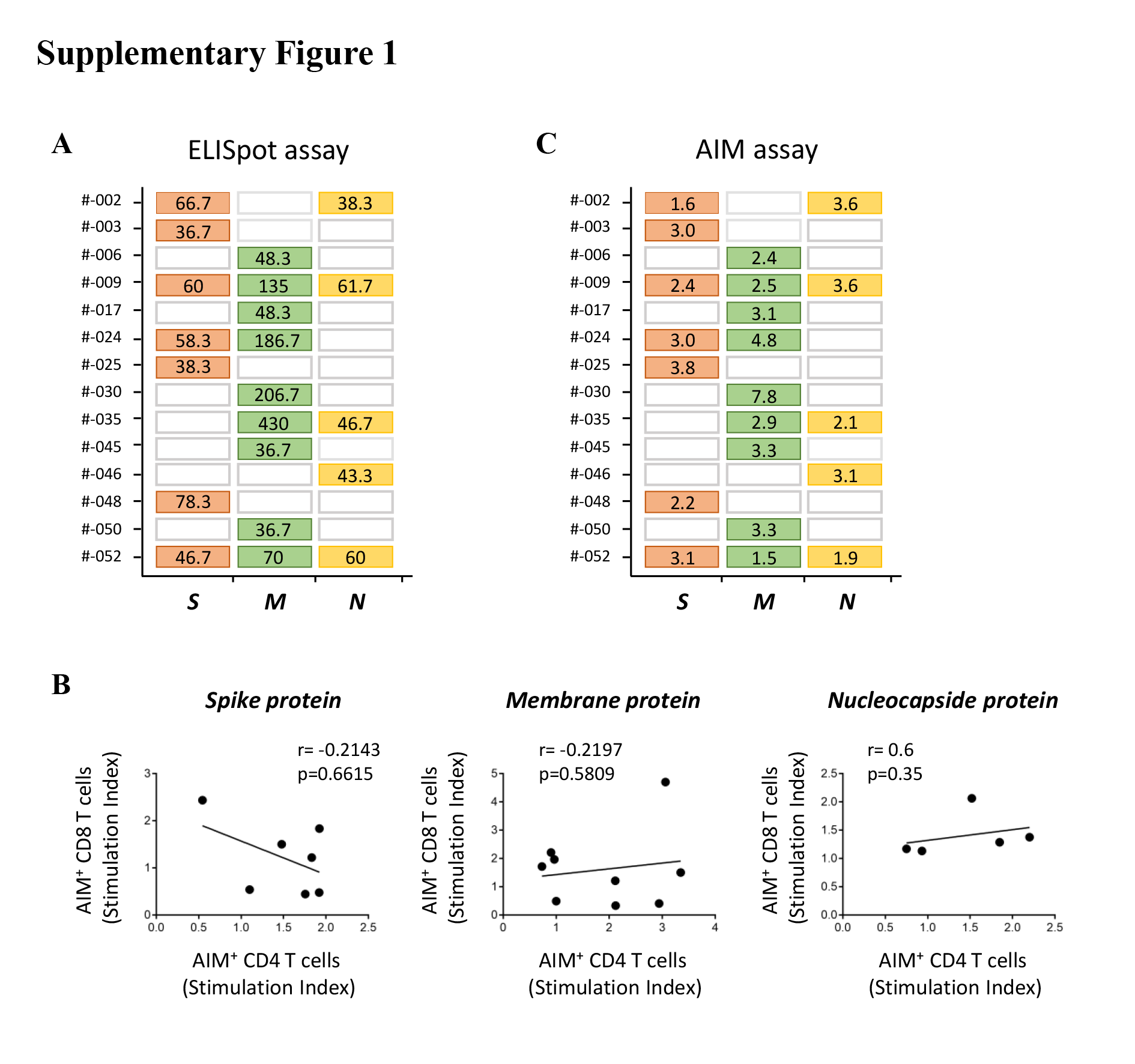

Supplement: Supplementary file 1 [file Image_1.tif]

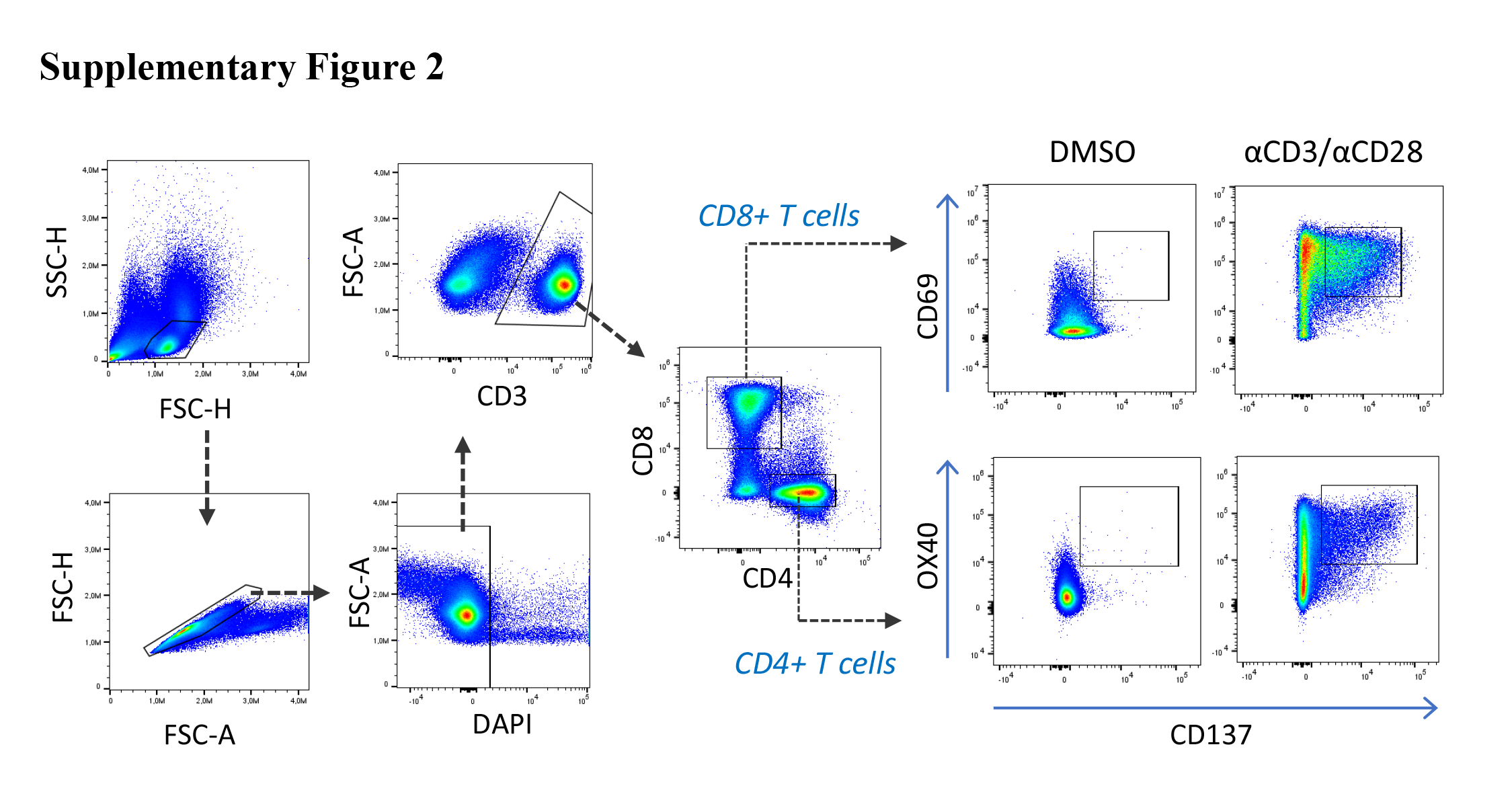

Supplement: Supplementary file 2 [file Image_2.tif]

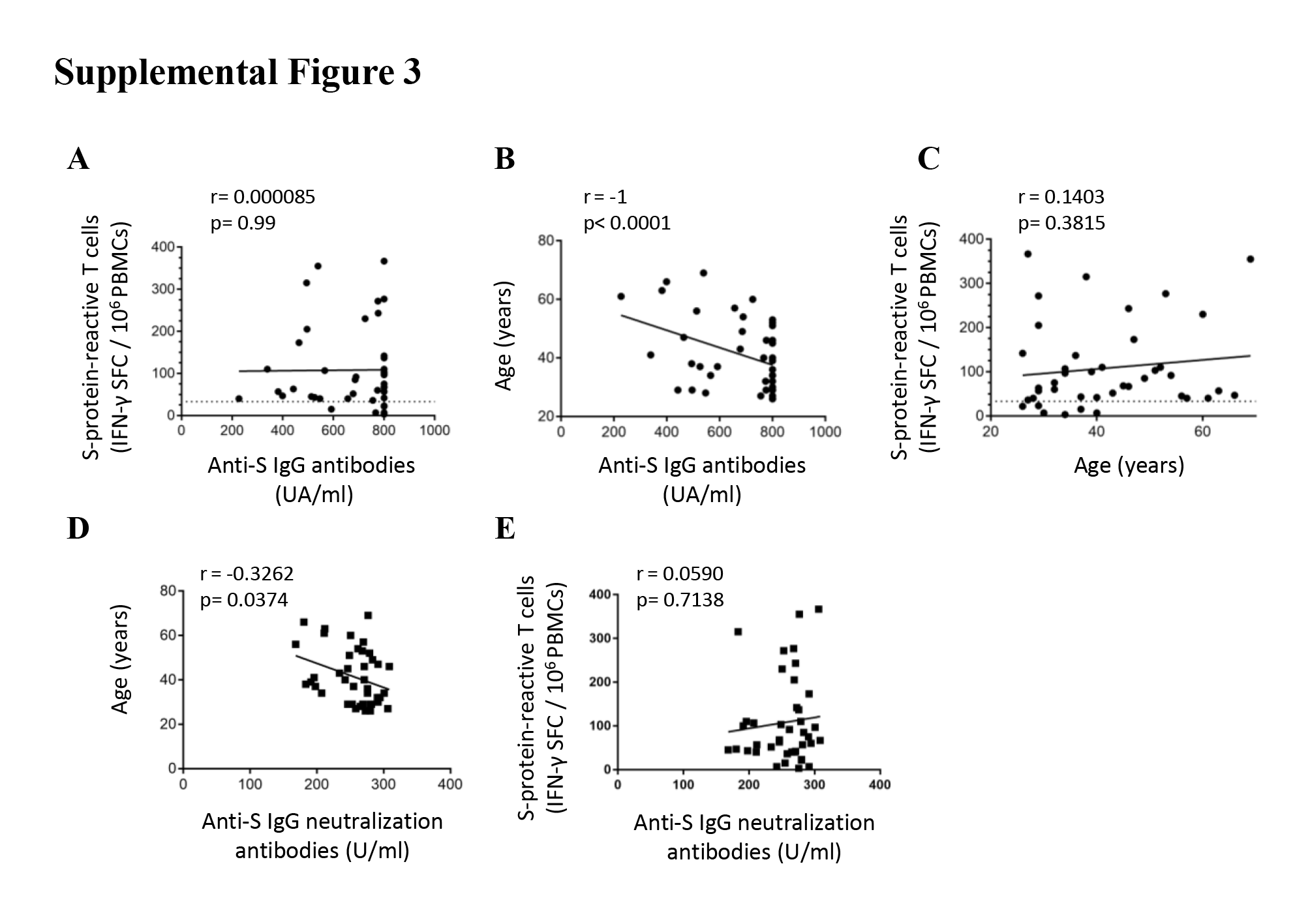

Supplement: Supplementary file 3 [file Image_3.tif]
